# Diagnostic Performance of AFP, PIVKA-II, and the ASAP Score for Distinguishing Hepatocellular Carcinoma With and Without Portal Vein Tumor Thrombus: A Retrospective Single-Center Study From Pakistan

**DOI:** 10.7759/cureus.114315

**Published:** 2026-08-10

**Authors:** Irtaza Ashraf, M Ejaz Khan, Syed Ali R Naqvi, M Babar Imran, Tania Jabbar

**Affiliations:** 1 Department of Chemistry, Government College University, Faisalabad, PAK; 2 Department of Oncology, Punjab Institute of Nuclear Medicine (PINUM) Cancer Hospital, Faisalabad, PAK; 3 Department of Nuclear Medicine, Punjab Institute of Nuclear Medicine (PINUM) Cancer Hospital, Faisalabad, PAK; 4 Department of Clinical Pathology, Punjab Institute of Nuclear Medicine (PINUM) Cancer Hospital, Faisalabad, PAK

**Keywords:** afp and pivka-ii, asap score, hepatocellular carcinoma, pakistani population, portal vein tumor thrombosis, serum tumor markers

## Abstract

Background: Portal vein tumor thrombus (PVTT) is an important manifestation of advanced hepatocellular carcinoma (HCC). Serum alpha-fetoprotein (AFP) and protein induced by vitamin K absence or antagonist-II (PIVKA-II) are established biomarkers associated with tumor burden, vascular invasion, and disease progression in HCC. However, their utility in distinguishing HCC patients with PVTT from those without PVTT remains poorly characterized in the Pakistani population.

Methods: This retrospective single-center study included 100 consecutive, newly diagnosed HCC patients: 46 with PVTT and 54 without PVTT. A separate group of 55 cancer-free controls was included solely for a secondary exploratory HCC-versus-control analysis. Diagnostic performance of AFP, PIVKA-II, and the Age-Sex-AFP-PIVKA-II (ASAP) score was evaluated using two-sided Mann-Whitney U tests, receiver operating characteristic (ROC) curve analysis, Youden index cutoffs, Spearman correlation, and multivariable logistic regression. ASAP values were calculated consistently from the raw variables before analysis.

Results: AFP and PIVKA-II were significantly higher in patients with PVTT than those without PVTT (AFP: p<0.001; PIVKA-II: p=0.030). AFP demonstrated the highest overall discriminatory performance for identifying PVTT, with an area under the ROC curve (AUROC) of 0.740 (95% confidence interval (CI): 0.642-0.837), achieving 78.3% sensitivity and 64.8% specificity at a cutoff value of 39.7 ng/mL. PIVKA-II yielded an AUROC of 0.627 (95% CI: 0.517-0.736), demonstrating high sensitivity (95.7%) but limited specificity (33.3%). The ASAP score achieved an AUROC of 0.688 (95% CI: 0.584-0.791) and did not outperform AFP. In multivariable logistic regression, natural logarithm-transformed (ln-transformed) AFP remained independently associated with PVTT (odds ratio: 1.249; 95% CI: 1.080-1.445; p=0.003), whereas ln-transformed PIVKA-II did not.

Conclusion: Among patients with established HCC, AFP demonstrated better overall discriminatory performance than PIVKA-II for identifying PVTT, whereas PIVKA-II provided high sensitivity but limited specificity. The ASAP score did not improve diagnostic performance beyond AFP alone. These biomarkers may complement, but should not replace, contrast-enhanced imaging for the assessment of PVTT. Larger prospective multicenter studies are required to validate these findings before routine clinical implementation.

## Introduction

Hepatocellular carcinoma (HCC) is one of the leading causes of cancer-related mortality worldwide, particularly in regions with a high prevalence of chronic hepatitis B virus (HBV) and hepatitis C virus (HCV) infections [1-3]. In Pakistan, the high prevalence of chronic viral hepatitis, delayed diagnosis, and limited access to specialized healthcare contribute substantially to the burden of advanced HCC [4].

Portal vein tumor thrombus (PVTT) is a clinically important manifestation of HCC progression and is associated with advanced tumor stage, restricted treatment options, and poor survival if left untreated [5-8]. Accurate identification of PVTT is essential because its presence influences disease staging, therapeutic decision-making, and prognosis. Although multiphasic contrast-enhanced computed tomography (CT) and magnetic resonance imaging (MRI) remain the reference standards for detecting PVTT, serum biomarkers may provide complementary information regarding tumor biology and vascular invasion.

Alpha-fetoprotein (AFP) remains the most widely used serum biomarker for HCC despite its recognized limitations in sensitivity and specificity across different stages of disease and underlying liver disorders [9,10]. Protein induced by vitamin K absence or antagonist-II (PIVKA-II), also known as des-γ-carboxy prothrombin, has been associated with tumor burden, vascular invasion, and aggressive tumor behavior [11-13]. Several studies have suggested that combining AFP and PIVKA-II may improve diagnostic performance for HCC; however, reported results vary across different populations and clinical settings [12-15].

The Age-Sex-AFP-PIVKA-II (ASAP) score was originally developed to detect HCC in patients with chronic liver disease by integrating demographic characteristics with serum biomarker measurements [16]. However, its role in distinguishing HCC patients with PVTT from those without PVTT has not been established. Therefore, application of the ASAP score in the present study should be considered exploratory rather than a validated clinical approach.

The primary objective of this study was to evaluate the diagnostic performance of AFP, PIVKA-II, and the ASAP score for distinguishing patients with HCC and PVTT from those without PVTT. Secondary objectives were to compare biomarker levels between HCC patients and a separate cancer-free control group and to explore the diagnostic performance of these biomarkers for differentiating HCC from cancer-free controls. No survival, recurrence, treatment-response, or prognostic outcomes were evaluated.

## Materials and methods

Study design and participants

This retrospective single-center observational study was conducted at the Punjab Institute of Nuclear Medicine (PINUM) in Faisalabad, Pakistan, between April 2024 and April 2025. The primary study cohort comprised 100 consecutive adult patients with newly diagnosed HCC. Based on radiological findings, patients were classified into two groups: group 1 included 46 patients with PVTT, and group 2 included 54 patients without PVTT. A secondary cohort of 55 cancer-free individuals with available AFP and PIVKA-II measurements and no radiological evidence of HCC during the study period was included solely for an exploratory HCC-versus-control analysis.

No formal sample size calculation was performed because this was a retrospective study, and all consecutive eligible patients who met the predefined inclusion criteria during the study period were included in the analysis.

The diagnosis of HCC was established using characteristic findings on multiphasic contrast-enhanced CT or MRI according to accepted international imaging criteria. PVTT status was determined from the final radiological assessment documented in the medical records. Tumor stage was classified according to the Eighth Edition of the American Joint Committee on Cancer (AJCC) TNM staging system. Because of the retrospective study design, detailed Liver Imaging Reporting and Data System (LI-RADS) tumor-in-vein descriptors, centralized blinded radiological review, and complete characterization of the control group were not consistently available.

Eligible patients were adults (aged ≥18 years) with newly diagnosed HCC who had AFP and PIVKA-II measured before the initiation of HCC-specific therapy. All patients underwent contrast-enhanced CT or MRI for tumor assessment and had complete clinical records for the variables included in the analysis. Patients were excluded if they had received previous treatment for HCC before biomarker assessment, had another active malignancy, were receiving vitamin K supplementation or anticoagulant therapy that could influence PIVKA-II concentrations, or had inadequate imaging for the determination of PVTT status.

The cancer-free controls were included exclusively for the secondary exploratory analysis and were not intended to represent a matched chronic liver disease surveillance population. Consequently, the results of this secondary analysis should be interpreted with appropriate caution.

Ethics approval

In accordance with institutional policy, retrospective record-based studies are exempt from formal ethical review. The PINUM Ethics Committee granted a waiver of ethical approval (letter number: PINUM-Estt-1(42)/2021). Only anonymized data extracted from existing medical records were analyzed, and no direct patient contact or intervention occurred. The study was conducted in accordance with the ethical principles of the Declaration of Helsinki.

Laboratory analysis

Peripheral venous blood samples (4 mL) were collected during the diagnostic evaluation and before the initiation of HCC-directed treatment. After clot formation, samples were centrifuged at 3,000 rpm for 10 minutes, and serum was separated for analysis. Serum AFP and PIVKA-II concentrations were measured using an electrochemiluminescence immunoassay (ECLIA) on the Roche COBAS Pro analytical platform (Roche Diagnostics, Rotkreuz, Switzerland) according to the manufacturer's calibration and quality control procedures.

ASAP score

The ASAP score was calculated using the published formula [16]: $\mathrm{ASAP}=&minus;7.58+(0.05\times\mathrm{Age})&minus;(0.58\times\mathrm{Sex})+(0.42\times\mathrm{ln[AFP]})+(1.11\times\mathrm{ln[PIVKA-II]})$

Age was entered in years, sex was coded as 0 for male and 1 for female, AFP was expressed in ng/mL, and PIVKA-II was expressed in mAU/mL. The ASAP score is dimensionless. Separate optimal cutoff values are reported because two different diagnostic questions were evaluated: (1) HCC with PVTT versus HCC without PVTT and (2) HCC versus cancer-free controls.

Statistical analysis

Statistical analyses were performed using IBM SPSS Statistics for Windows, V. 23.0 (IBM Corp., Armonk, NY, USA), and independently verified using Python statistical libraries (Python Software Foundation, Wilmington, DE, USA).

Normality was assessed using the Kolmogorov-Smirnov and Shapiro-Wilk tests. Because continuous variables were not normally distributed, they are presented as median and interquartile range (IQR). Comparisons between HCC patients with and without PVTT were performed using the two-sided Mann-Whitney U test for continuous variables and the chi-squared test for categorical variables.

Receiver operating characteristic (ROC) curve analysis was used to evaluate the diagnostic performance of AFP, PIVKA-II, and the ASAP score. Areas under the ROC curve (AUROCs) with 95% confidence intervals (CIs) were calculated, and optimal cutoff values were determined using Youden's index. Sensitivity, specificity, positive predictive value (PPV), negative predictive value (NPV), and diagnostic accuracy were calculated from the corresponding confusion matrices.

Spearman rank correlation analysis was used to evaluate associations between biomarker levels and PVTT status. Multivariable binary logistic regression was performed using natural logarithm-transformed (ln-transformed) AFP, ln-transformed PIVKA-II, age, and sex as prespecified covariates. Adjusted odds ratios (ORs) with 95% CIs are reported. All statistical tests were two-sided, and a p-value of <0.05 was considered statistically significant.

## Results

The primary study cohort comprised 100 consecutive patients with newly diagnosed HCC, including 46 patients with PVTT and 54 patients without PVTT. A secondary exploratory cohort consisted of 55 cancer-free controls.

The median age did not differ significantly between patients with PVTT and those without PVTT (58.0 years (IQR: 54.3-62.8) vs. 60.0 years (IQR: 50.0-65.0); p=0.827). However, male patients were significantly more common in the PVTT group than in the non-PVTT group (80.4% vs. 59.3%; p=0.022). As expected, all patients with PVTT had advanced disease (AJCC stage III or IV), whereas patients without PVTT were distributed across stages I-IV (Table 1).

**Table 1:** Demographic and clinical characteristics. The primary p-values compare HCC with PVTT versus HCC without PVTT. Continuous variables are reported as median (IQR). IQR: interquartile range; HCC: hepatocellular carcinoma; PVTT: portal vein tumor thrombus

Characteristic	HCC with PVTT (n=46)	HCC without PVTT (n=54)	Cancer-free controls (n=55)	Primary p-value
Age, years, median (IQR)	58.0 (54.3-62.8)	60.0 (50.0-65.0)	54.0 (49.0-60.5)	0.827
Sex, n (%)				
Male	37 (80.4)	32 (59.3)	29 (52.7)	0.022
Female	9 (19.6)	22 (40.7)	26 (47.3)	
TNM stage, n (%)				
I	0	2 (3.7)	Not applicable	Not tested
II	0	19 (35.2)	Not applicable	
III	30 (65.2)	15 (27.8)	Not applicable	
IV	16 (34.8)	18 (33.3)	Not applicable	

Serum AFP, PIVKA-II, and ASAP scores demonstrated non-normal distributions and are therefore presented as medians with IQR. Median AFP concentrations were significantly higher in patients with PVTT than in those without PVTT (696.0 ng/mL (IQR: 46.3-6938.0) vs. 13.35 ng/mL (IQR: 4.59-551.03); p<0.001). Similarly, median PIVKA-II concentrations were significantly higher in the PVTT group than in the non-PVTT group (144.5 mAU/mL (IQR: 37.3-1112.1) vs. 49.0 mAU/mL (IQR: 2.0-257.0); p=0.030).

The median ASAP score was also significantly higher among patients with PVTT than among those without PVTT (3.75 (IQR: 1.61-6.70) vs. 0.98 (IQR: −1.61-4.13); p=0.001). In the secondary exploratory analysis, all three biomarkers were significantly higher in HCC patients than in cancer-free controls (all p<0.001) (Table 2).

**Table 2:** Biomarker distributions in the study cohort. The primary p-value compares HCC with versus without PVTT. The secondary p-value compares all HCC patients with cancer-free controls using a two-sided Mann-Whitney U test. IQR: interquartile range; HCC: hepatocellular carcinoma; PVTT: portal vein tumor thrombus

Marker	HCC with PVTT median (IQR)	HCC without PVTT median (IQR)	Cancer-free controls median (IQR)	Primary p-value	Secondary p-value
AFP (ng/mL)	696.0 (46.3-6938.0)	13.35 (4.59-551.03)	1.90 (1.32-3.66)	<0.001	<0.001
PIVKA-II (mAU/mL)	144.5 (37.3-1112.1)	49.0 (2.0-257.0)	18.7 (15.2-21.4)	0.030	<0.001
ASAP score	3.75 (1.61-6.70)	0.98 (-1.61-4.13)	-1.74 (-2.09 to -1.06)	0.001	<0.001

**Figure 1:**
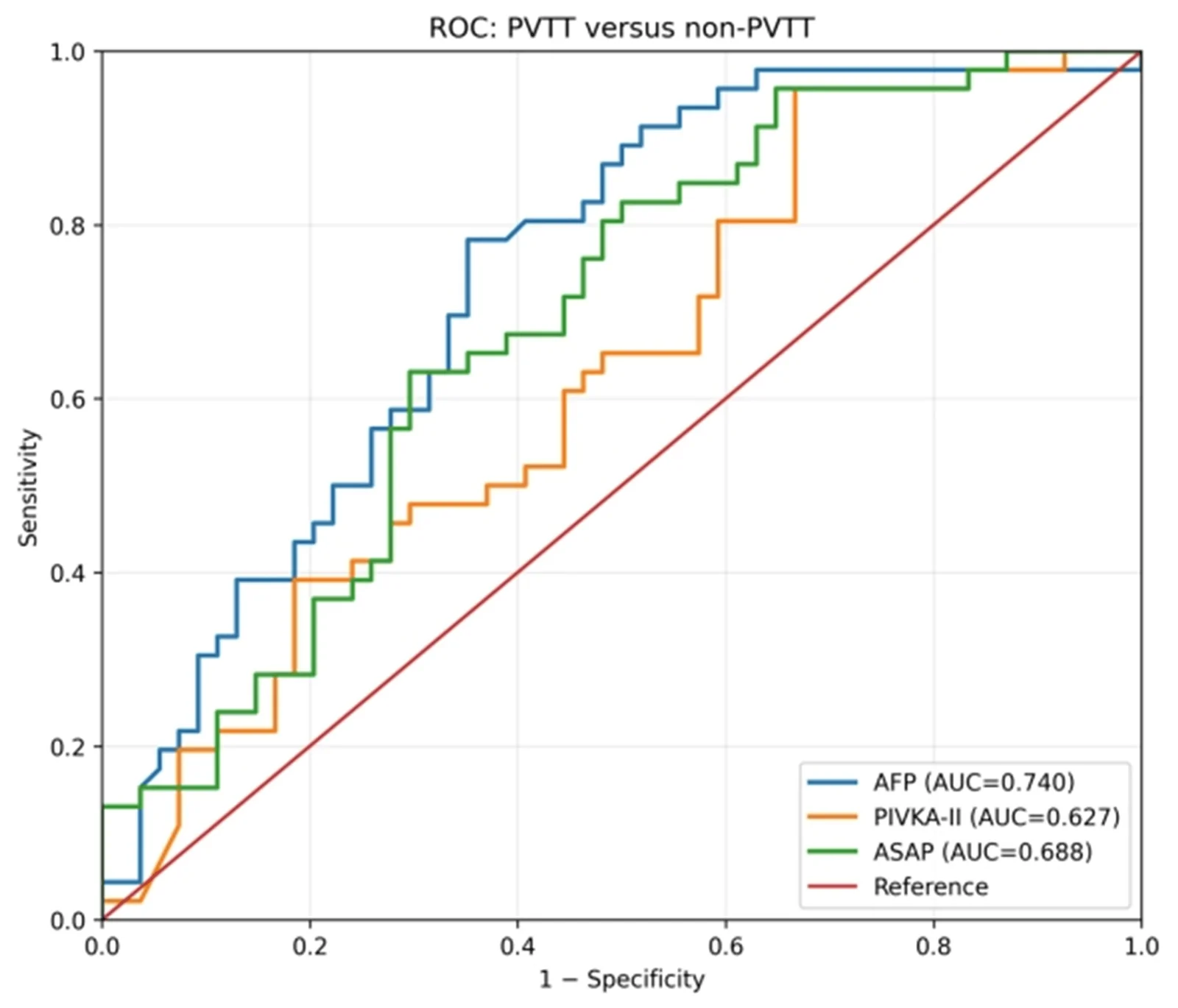
ROC curves for AFP, PIVKA-II, and the ASAP score in the primary comparison of HCC with PVTT versus HCC without PVTT. HCC: hepatocellular carcinoma; PVTT: portal vein tumor thrombus; AFP: alpha-fetoprotein; PIVKA-II: protein induced by vitamin K absence or antagonist-II; ASAP: Age-Sex-AFP-PIVKA-II; ROC: receiver operating characteristic; AUC: area under the curve

ROC curve analysis was performed to evaluate the diagnostic performance of AFP, PIVKA-II, and the ASAP score for distinguishing HCC patients with PVTT from those without PVTT (Figure 1).

AFP demonstrated the highest overall discriminatory performance, with an AUROC of 0.740 (95% CI: 0.642-0.837; p<0.001). At the optimal cutoff value of 39.7 ng/mL, AFP achieved a sensitivity of 78.3%, a specificity of 64.8%, a PPV of 65.5%, an NPV of 77.8%, and an overall diagnostic accuracy of 71.0%.

PIVKA-II demonstrated an AUROC of 0.627 (95% CI: 0.517-0.736; p=0.023). Using a cutoff value of 9.59 mAU/mL, PIVKA-II achieved high sensitivity (95.7%) but relatively low specificity (33.3%), resulting in an overall diagnostic accuracy of 62.0%.

The ASAP score showed intermediate diagnostic performance, with an AUROC of 0.688 (95% CI: 0.584-0.791; p<0.001). At the optimal cutoff value of 2.897, the ASAP score achieved 63.0% sensitivity, 70.4% specificity, and an overall diagnostic accuracy of 67.0% (Table 3).

**Table 3:** Primary diagnostic performance for distinguishing HCC with PVTT from HCC without PVTT. AUROC: area under the ROC curve; HCC: hepatocellular carcinoma; PVTT: portal vein tumor thrombus; AFP: alpha-fetoprotein; PIVKA-II: protein induced by vitamin K absence or antagonist-II; ASAP: Age-Sex-AFP-PIVKA-II; PPV: positive predictive value; NPV: negative predictive value

Marker	AUROC (95% CI)	Cutoff	Sensitivity/specificity (%)	PPV/NPV (%)	Accuracy (%)	Youden J
AFP	0.740 (0.642-0.837)	39.7 ng/mL	78.3/64.8	65.5/77.8	71.0	0.431
PIVKA-II	0.627 (0.517-0.736)	9.59 mAU/mL	95.7/33.3	55.0/90.0	62.0	0.290
ASAP	0.688 (0.584-0.791)	2.897	63.0/70.4	64.4/69.1	67.0	0.334

Spearman rank correlation analysis demonstrated statistically significant positive correlations between PVTT status and AFP (ρ=0.414; p<0.001), PIVKA-II (ρ=0.219; p=0.029), and the ASAP score (ρ=0.324; p=0.001), indicating that higher biomarker concentrations were associated with the presence of PVTT.

Multivariable logistic regression

Multivariable binary logistic regression was performed using ln-transformed AFP, ln-transformed PIVKA-II, age, and sex as prespecified covariates.

After adjustment for the remaining variables, ln-transformed AFP remained independently associated with PVTT (adjusted OR: 1.249; 95% CI: 1.080-1.445; p=0.003). In contrast, ln-transformed PIVKA-II (adjusted OR: 1.097; 95% CI: 0.955-1.259; p=0.189), age (p=0.382), and sex (p=0.205) were not independently associated with PVTT (Table 4).

**Table 4:** Multivariable logistic regression for PVTT status among HCC patients. Outcome coding: PVTT=1; non-PVTT=0. AFP: alpha-fetoprotein; PIVKA-II: protein induced by vitamin K absence or antagonist-II; HCC: hepatocellular carcinoma; PVTT: portal vein tumor thrombus; ln-transformed: natural logarithm-transformed

Variable	Adjusted OR	95% CI	P-value
ln-transformed AFP	1.249	1.080-1.445	0.003
ln-transformed PIVKA-II	1.097	0.955-1.259	0.189
Age, per year	1.027	0.968-1.089	0.382
Female versus male	0.524	0.193-1.422	0.205

**Figure 2:**
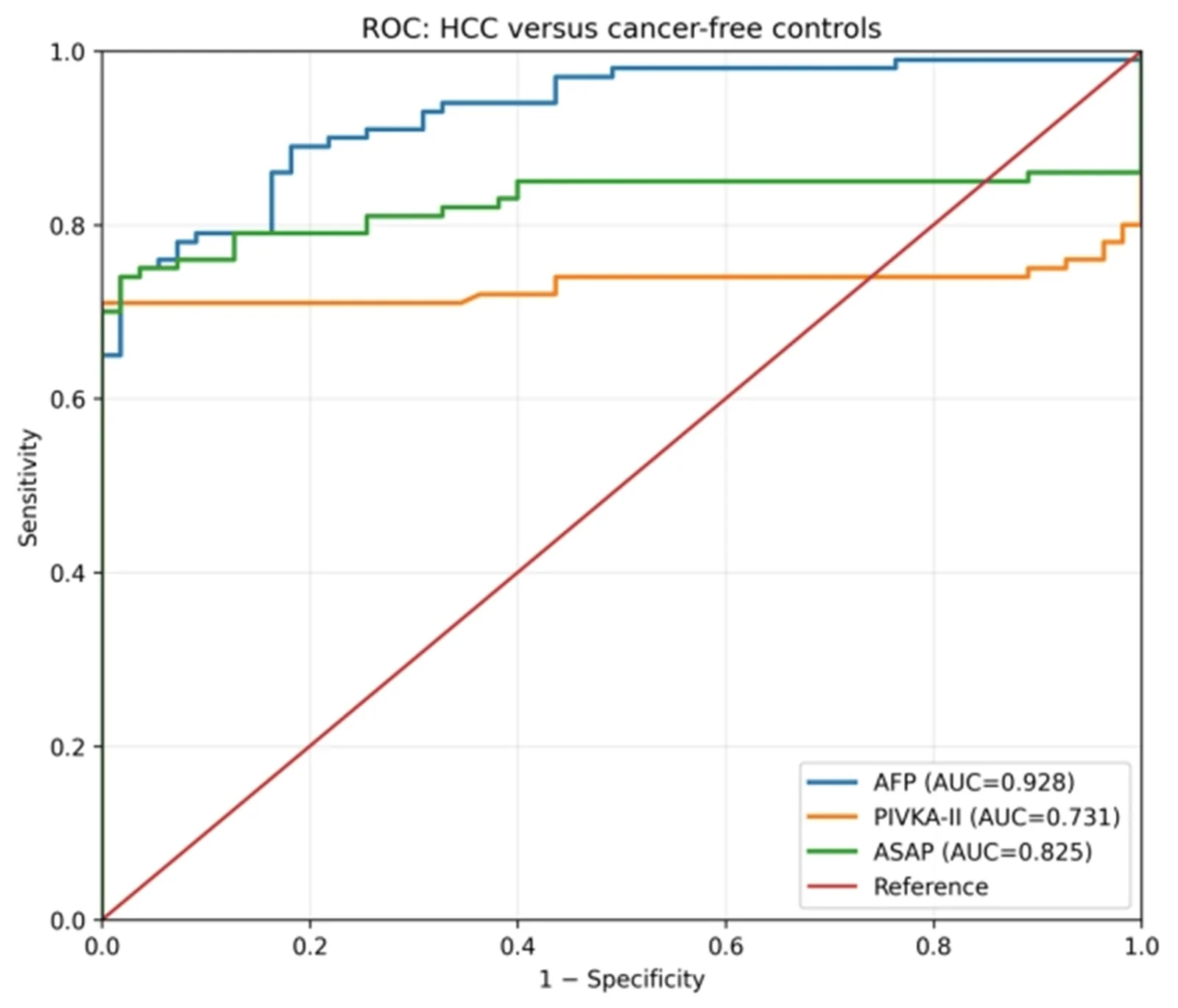
ROC curves for AFP, PIVKA-II, and the ASAP score in the secondary exploratory comparison of all HCC patients versus cancer-free controls. HCC: hepatocellular carcinoma; AFP: alpha-fetoprotein; PIVKA-II: protein induced by vitamin K absence or antagonist-II; ASAP: Age-Sex-AFP-PIVKA-II; ROC: receiver operating characteristic; AUC: area under the curve

A secondary ROC curve analysis compared all HCC patients with the cancer-free control group (Figure 2).

AFP demonstrated excellent diagnostic performance, with an AUROC of 0.928 (95% CI: 0.890-0.967), followed by the ASAP score (AUROC: 0.825) and PIVKA-II (AUROC: 0.731). Because the control group did not represent a matched chronic liver disease surveillance population, these findings should be interpreted as exploratory and should not be directly compared with the primary PVTT analysis (Table 5).

**Table 5:** Secondary exploratory performance for differentiating HCC patients from cancer-free controls. These estimates are not directly comparable with the primary PVTT analysis and may overestimate specificity because the controls were not a matched chronic liver disease population. HCC: hepatocellular carcinoma; AFP: alpha-fetoprotein; PIVKA-II: protein induced by vitamin K absence or antagonist-II; ASAP: Age-Sex-AFP-PIVKA-II; PPV: positive predictive value; NPV: negative predictive value; AUROC: area under the ROC curve; PVTT: portal vein tumor thrombus

Marker	AUROC (95% CI)	Cutoff	Sensitivity/specificity (%)	PPV/NPV (%)	Accuracy (%)
AFP	0.928 (0.890-0.967)	7.076 ng/mL	74.0/98.2	98.7/67.5	82.6
PIVKA-II	0.731 (0.645-0.816)	29.466 mAU/mL	71.0/100.0	100.0/65.5	81.3
ASAP	0.825 (0.755-0.896)	-0.212	74.0/98.2	98.7/67.5	82.6

## Discussion

The present study evaluated the diagnostic performance of AFP, PIVKA-II, and the ASAP score for distinguishing HCC patients with PVTT from those without PVTT. Three principal findings emerged. First, serum AFP, PIVKA-II, and ASAP scores were significantly higher in patients with PVTT than in those without PVTT. Second, AFP demonstrated the highest overall discriminatory performance for identifying PVTT, whereas PIVKA-II achieved the highest sensitivity but relatively low specificity. Third, the ASAP score demonstrated intermediate diagnostic performance and did not improve discrimination beyond AFP alone.

PVTT is a well-recognized manifestation of advanced HCC and is associated with increased tumor aggressiveness, restricted therapeutic options, and poor prognosis. Accurate identification of PVTT is therefore essential for disease staging, treatment planning, and prognostic assessment. Although contrast-enhanced CT and MRI remain the reference standards for detecting PVTT, serum biomarkers may provide complementary information regarding tumor biology and vascular invasion.

AFP remains the most widely used serum biomarker for HCC despite its recognized limitations in sensitivity and specificity. In the present study, AFP demonstrated the highest AUROC (0.740) among the evaluated biomarkers and remained independently associated with PVTT after adjustment for age, sex, and PIVKA-II. These findings suggest that AFP continues to provide useful discrimination for vascular invasion in patients with established HCC. However, AFP should not be considered a standalone diagnostic marker for PVTT because false-negative results may occur, particularly in biologically heterogeneous tumors.

PIVKA-II has been increasingly recognized as a biomarker associated with tumor burden, vascular invasion, and aggressive tumor biology. In agreement with previous studies, serum PIVKA-II concentrations were significantly higher in patients with PVTT than in those without PVTT. Although PIVKA-II demonstrated excellent sensitivity (95.7%) at the selected cutoff value, its specificity was limited (33.3%), resulting in a relatively high false-positive rate. These findings suggest that PIVKA-II may be more useful as a sensitive complementary biomarker than as an independent diagnostic test for PVTT.

The ASAP score was originally developed for the detection of HCC in patients with chronic liver disease rather than for differentiating PVTT status among patients with established HCC. In the present study, the ASAP score demonstrated intermediate diagnostic performance (AUROC 0.688) and did not outperform AFP alone. Therefore, the use of the ASAP score for PVTT classification should currently be considered exploratory, and additional external validation is required before clinical implementation in this setting.

Comparison with previous studies should be interpreted cautiously because differences in study populations, underlying liver disease etiology, assay methodology, biomarker cutoff values, and disease stage may substantially influence diagnostic performance. Several international studies have demonstrated that AFP and PIVKA-II are valuable biomarkers for HCC diagnosis. PIVKA-II showed excellent diagnostic performance for HBV-related HCC in a large multicenter Chinese cohort, while Park et al. demonstrated that combining AFP, AFP-L3, and PIVKA-II improved diagnostic accuracy compared with individual biomarkers alone [14]. Similarly, Feng et al., Loglio et al., and Omata et al. found that the combination of AFP and PIVKA-II provided better diagnostic performance than either biomarker alone in different patient populations [12,15,17]. Furthermore, Sun et al. demonstrated that the ASAP score performed well for detecting HCC in patients with chronic liver disease, supporting its role as a diagnostic model for HCC rather than for PVTT differentiation [16].

However, most of these studies primarily evaluated the diagnosis of HCC based on (AJCC) TNM staging system [18], whereas comparatively few specifically investigated the ability of these biomarkers to distinguish HCC patients with and without PVTT. In our study, both AFP and PIVKA-II concentrations were significantly higher in patients with PVTT than in those without PVTT. Following comprehensive statistical reanalysis, AFP demonstrated the highest overall discriminatory performance for PVTT differentiation, whereas PIVKA-II showed higher sensitivity but lower specificity. The ASAP score did not outperform AFP alone and should therefore be regarded as an exploratory model in this clinical setting rather than a validated tool for PVTT detection. Consequently, the present study complements existing international evidence by providing data from a Pakistani population, where published studies evaluating biomarker performance specifically for PVTT remain limited.

Strengths and limitations

This study has several strengths. It represents one of the few studies from Pakistan evaluating AFP, PIVKA-II, and the ASAP score specifically in relation to PVTT. Consecutive patient inclusion reduced the likelihood of selection bias, biomarker measurements were obtained before the initiation of HCC-directed therapy, and the primary and secondary analyses were clearly separated to address distinct clinical questions.

Several limitations should also be acknowledged. First, this was a retrospective single-center study with a relatively modest sample size, which may limit the generalizability of the findings. Second, no formal sample size calculation was performed because all eligible consecutive patients during the study period were included. Third, the control group was not a matched chronic liver disease cohort, potentially influencing the exploratory HCC-versus-control analysis. Fourth, important clinical variables, including Child-Pugh class, Model for End-Stage Liver Disease (MELD) score, tumor size, tumor multiplicity, viral etiology, liver function parameters, and treatment outcomes, were not consistently available and therefore could not be incorporated into the multivariable analysis. Fifth, PVTT classification was based on routine radiological reports without centralized blinded image review. Finally, because no formal statistical comparison between ROC curves (e.g., DeLong testing) was performed, differences in AUROC values should be interpreted descriptively rather than as evidence of statistically superior diagnostic performance.

## Conclusions

In this retrospective single-center study, serum AFP, PIVKA-II, and the ASAP score were significantly higher in patients with HCC and PVTT than in those without PVTT. Among the evaluated biomarkers, AFP demonstrated the best overall discriminatory performance for identifying PVTT, whereas PIVKA-II provided high sensitivity but limited specificity. The ASAP score showed intermediate diagnostic performance and did not improve discrimination beyond AFP alone.

These findings suggest that AFP and PIVKA-II may provide complementary information in the evaluation of patients with established HCC when interpreted alongside clinical and radiological findings. However, none of the evaluated biomarkers demonstrated sufficient diagnostic performance to replace contrast-enhanced imaging for the assessment of PVTT.

Given the retrospective single-center design and the relatively modest sample size, these findings should be interpreted with appropriate caution. Larger prospective multicenter studies are needed to validate the diagnostic utility of AFP, PIVKA-II, and the ASAP score before their routine clinical application for PVTT assessment.
